# Quantitative, in situ analysis of mRNAs and proteins with subcellular resolution

**DOI:** 10.1038/s41598-017-16492-1

**Published:** 2017-11-28

**Authors:** Sunjong Kwon, Koei Chin, Michel Nederlof, Joe W. Gray

**Affiliations:** 1 Department of Biomedical Engineering, OHSU Center for Spatial Systems Biomedicine, Oregon Health & Science University, 2730 SW Moody Ave, Portland, OR 97201 USA; 2 Quantitative Imaging Systems, Inc., 1502 Fox Chapel Road, Pittsburgh, PA 15238 USA

## Abstract

We describe here a method, termed immunoFISH, for simultaneous *in situ* analysis of the composition and distribution of proteins and individual RNA transcripts in single cells. Individual RNA molecules are labeled by hybridization and target proteins are concurrently stained using immunofluorescence. Multicolor fluorescence images are acquired and analyzed to determine the abundance, composition, and distribution of hybridized probes and immunofluorescence. We assessed the ability of immunoFISH to simultaneous quantify protein and transcript levels and distribution in cultured HER2 positive breast cancer cells and human breast tumor samples. We demonstrated the utility of this assay in several applications including demonstration of the existence of a layer of normal myoepithelial KRT14 expressing cells that separate HER2+ cancer cells from the stromal and immune microenvironment in HER2+ invasive breast cancer. Our studies show that immunoFISH provides quantitative information about the spatial heterogeneity in transcriptional and proteomic features that exist between and within cells.

## Introduction

The abundance, composition and spatial distribution of diverse RNA transcripts and proteins are fundamental determinants of the behavior of normal and cancer cells and numerous technologies have been developed to measure these cellular features. In the past decade, work has focused on the development of ‘omic methods such as mass spectrometry and massively parallel sequencing that enable comprehensive and quantitative measures of protein and transcript profiles^1,2^. These methods have enabled discovery of important functional components of normal cells and have revealed changes in specific transcripts and proteins from normal that suggest mechanisms by which diseases arise, progress and respond to treatment. These analyses also have guided the development of new therapeutic approaches and diagnostic assays and stimulated the current interest in precision medicine. However, ‘omic analysis methods provide little information about the rich and functionally important variations in transcriptional and proteomic features that exist between and within cells. In cancer, for example, variation between cells is caused by genomic and epigenomic instability^3–5^ and variability in signals from the diverse microenvironments in which the cancer cells reside^6–9^. These differences may translate into differences in biological behavior, metastatic potential^10^ and therapeutic sensitivity^11^. Assessment of the locations of specific mRNA transcripts and proteins within cells allows study of a variety of cancer related processes; for example, nuclear processing of nascent pre-messenger RNA (pre-mRNA) transcripts, alternative splicing and receptor recycling following treatment with targeted therapeutics and the spatial organization of molecularly distinct cells within tissues.

We show in this paper that simultaneous *in situ* measurement of transcripts location and composition and protein levels is possible in most circumstances by combining aspects of RNA fluorescence *in situ* hybridization (FISH) and immunofluorescence staining. In our approach, hereafter referred to as immunoFISH, we detect specific transcripts using synthetic oligonucleotide probes labeled with bright fluorophores that target multiple regions along the desired transcripts^12–14^ and specific proteins by immunofluorescent staining. Signals from fluorescently labeled proteins and individual transcripts are quantified by analysis of images acquired using high-resolution fluorescence microscopy. This approach builds on previous reports showing that coding and noncoding RNA transcripts can be quantified and spatially localized^15,16^ and even sequenced *in situ* ^17^.

We demonstrate the utility of immunoFISH by assessing the location and abundance of HER2 introns and exons relative to the nuclear boundary defined by immunostaining for nuclear lamins and HER2 protein in cultured cancer cell lines and formalin fixed paraffin embedded tissue. The quantitative nature of immunoFISH was established by comparing mRNA and protein levels measured using immunoFISH with levels measured using RNA-seq and western blotting, respectively. We also applied immunoFISH for temporal analysis of HER2 mRNA and AKT1 mRNA expression, and phosphoAKT protein levels in HER2-positive breast cancer single cells treated with the HER-family tyrosine kinase inhibitor lapatinib. Finally, we used immunoFISH to assess KRT14 and HER2 protein levels and HER2 mRNA expression in formal-fixed, paraffin embedded tissues and showed the remarkable spatial heterogeneity that exists within individual HER2+ cancers.

## Results

### Quantitative immunoFISH

**Figure 1:**
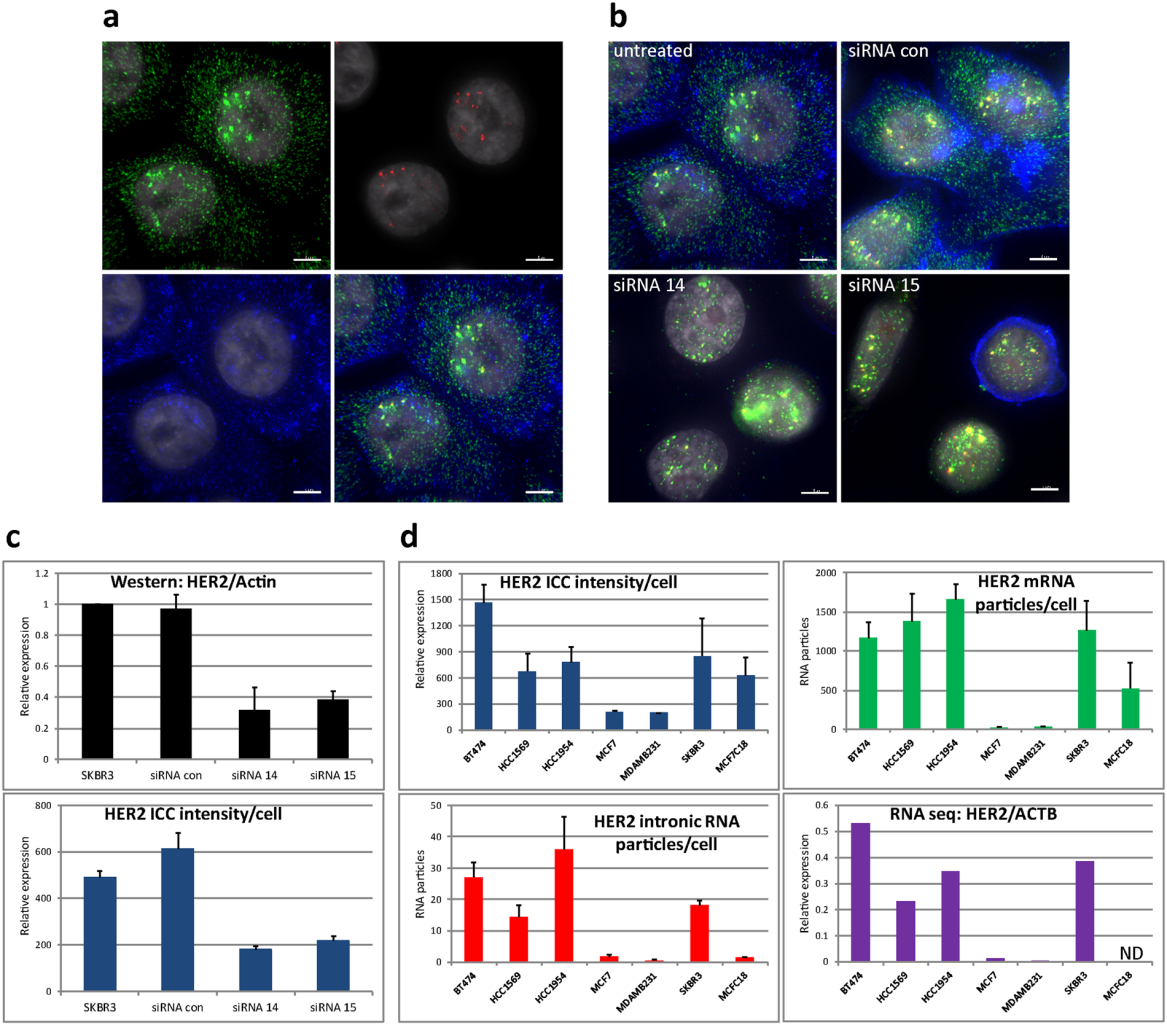
Quantitative analysis of HER2 mRNA and protein levels in breast cancer cell lines by immunoFISH. (**a**) Simultaneous co-imaging of HER2 proteins (blue), HER2 mRNAs (green), intronic RNAs (red), and nuclei (gray) on SKBR3 breast cancer cells. All intronic RNA particles are located in nuclei, overlapping with exonic RNA particles (yellow) in merged image. The whole-field view images are presented in Supplementary Fig. S5. (**b**) HER2 immunoFISH on SKBR3 with HER2 knock-down by two different siRNAs, siRNA14 and siRNA15. Cytoplasmic particles of exonic RNAs (green) are gone but nuclear particles of exonic (green and yellow) and intronic RNAs (yellow) remain intact. The whole-field view images are presented in Supplementary Fig. S6. (**c**) Comparison of HER2 protein levels by western blotting and immunoFISH of HER2 knock-down by siRNAs. Relative levels of HER2 protein and actin protein of knock-down by siRNAs were quantified based on band intensity on western blots from three different experiments. HER2 protein intensities in each cell of immunoFISH were measured by mean of pixel intensities of HER2 Ab staining channel from segmented cells using Qi Tissue software. (**d**) Comparison of HER2 mRNA and proteins levels by immunoFISH and RNA-seq of breast cancer cell lines. The number of total RNA spots in a reconstructed 3D image was counted by IMARIS, and then the total was divided by the number of nuclei to obtain the number of transcripts in single cells. For RNA-seq data^21^, raw read counts of HER2 were divided by those of beta-actin. ND = not determined. Bar is 5 μm.

We assessed the performance of immunoFISH for quantitative analysis of cellular expression of mRNA and protein by comparing levels measured using immunoFISH with those measured using RNA-seq and western blot analysis. These measurements were carried out by analyzing breast cancer cell lines that varied in the levels of expression of HER2 mRNA and protein. We assessed HER2 mRNA composition by hybridizing with probes separately targeting the first 4 introns after the start cordon and/or 25 exons encoding whole open reading frame of HER2 protein (NCBI Reference Sequence: NM_001005862.1). The intron and exon probes were labeled with CAL Fluor Red 610 and Quasar670 fluorophores, respectively, so that their locations could be visualized separately. Figure 1a shows ImmunoFISH images of HER2 exonic and intronic transcripts, and protein in the HER2-amplified breast cancer cell line, SKBR3. Similar results were found for the HER2 amplified cell lines BT474, HCC1954, HCC1569, and MCF7C18 (Supplementary Fig. S1). Figure 1b shows results for SKBR3 cells treated with a short interfering RNA targeting HER2 (siHER2). In this case, the cytoplasmic transcripts are gone but the nuclear transcripts remain. This is consistent with the known cytoplasmic location of the RNA-induced silencing complex (RISC)^18,19^. Quantitative image analysis of individual mRNA transcripts showed ~1300 HER2 mRNA hybridization signals in the cytoplasm and nucleus of control cells and about an eighth this number located only in the nuclei of cells treated with the siRNA against HER2 in SKBR3 cells (Supplementary Fig. S2). Figure 1c shows concordance between HER2 levels measured using western blotting and by quantifying HER2 levels via analysis of images acquired after immunofluorescence staining for HER2.

We further assessed the quantitative performance of immunoFISH by analyzing HER2 transcript and protein levels in 7 different breast cancer cell lines including 4 in which HER2 was amplified^20^. We compared these levels to protein and transcript levels determined using quantitative western blotting and RNA-seq^21^, respectively. We measured the number of HER2 mRNA particles per cell and measured total HER2 protein staining intensity levels during immunoFISH in ~40 cells. We found that the average number of HER2 transcripts per cell correlated closely with the number of HER2 transcripts measured using RNA-seq (Fig. 1d) relative to GAPDH (*r* = 0.84) or beta-actin values (*r* = 0.81). We also found that the average levels of HER2 immunofluorescence per cell correlated well (*r* = 0.82) with levels of HER2 protein relative to actin protein measured using western blotting (Supplementary Fig. S3).

### Intracellular distribution and composition of HER2 mRNA

We further explored the ability of immunoFISH to inform on the intracellular locations of RNA species and compositions by assessing intronic and exonic HER2 mRNA transcripts in cells that had been immunofluorescently stained for nuclear lamin A/C to define nuclear boundaries. Nuclear lamins are building blocks of nuclear architecture, and A/C lamins assemble on the inside of the nuclear envelope^22^.

**Figure 2:**
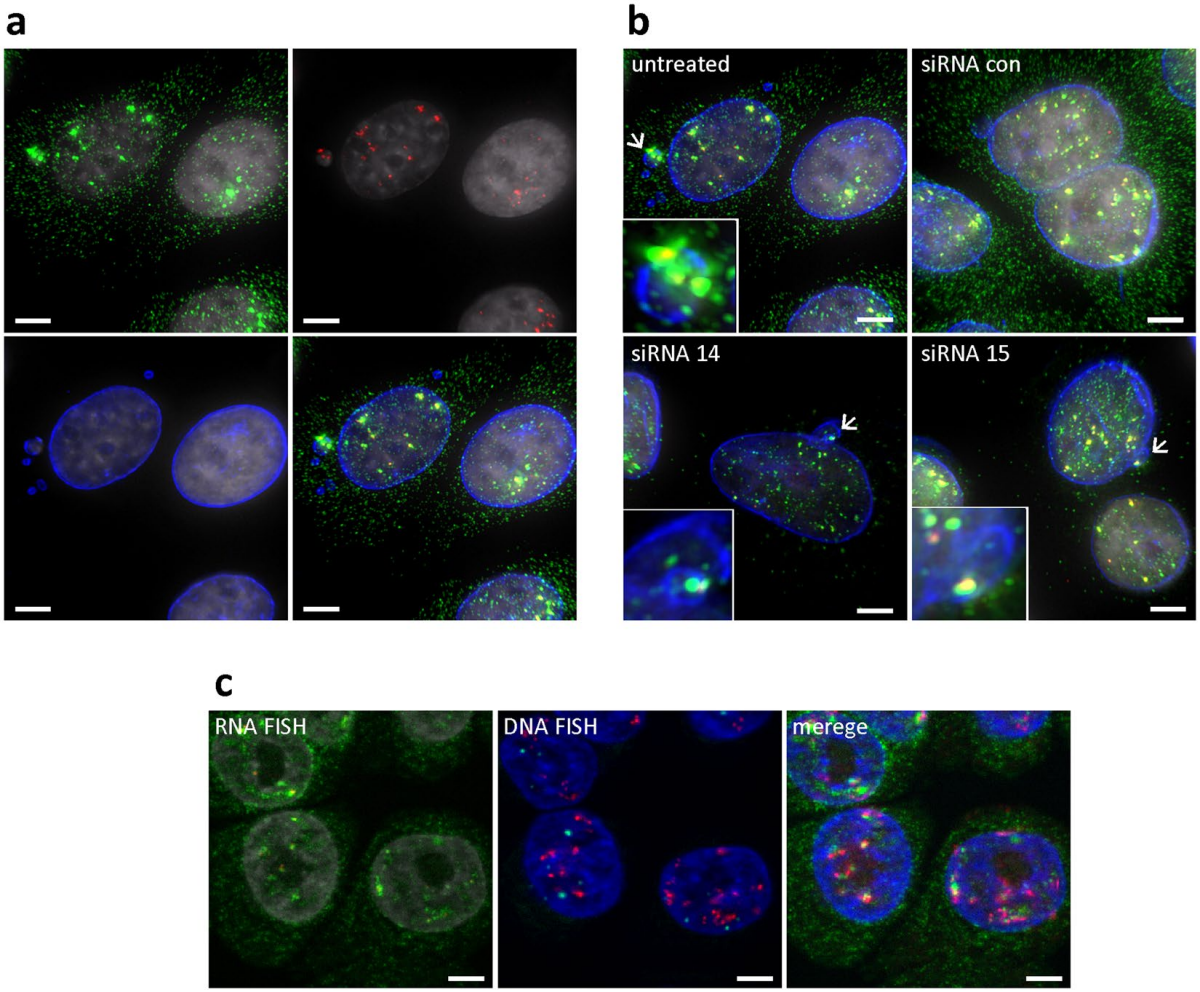
Analysis of HER2 mRNA subcellular location by immunoFISH. (**a**) ImmunoFISH for nuclear lamin A/C protein (blue), HER2 mRNAs (green), intronic RNAs (red), and nuclei (gray) on SKBR3 cells. Intron containing HER2 mRNA signals are colocalized with exon containing HER2 mRNA, suggesting colocalized particles (yellow) are HER2 pre-mRNAs. The whole-field view images are presented in Supplementary Fig. S7. (**b**) ImmunoFISH for lamin A/C protein and HER2 mRNA on SKBR3 with HER2 knock-down by siRNAs. Intron containing HER2 mRNAs are exclusively detected in nuclei and micronuclei (arrows) suggesting HER2 pre-mRNAs are transcribed not only in nuclei but also in micronuclei. The whole-field view images are presented in Supplementary Fig. S8. (**c**) Consecutive HER2 RNA FISH (HER2 mRNAs – green; intronic RNAs – red; nuclei – gray) and DNA FISH (copy control 17– green; HER2 – red; nuclei – blue). Please note that nuclei become slightly shrunk during harsh DNA FISH condition, and nuclei from DNA FISH were registered aligning to those from RNA FISH. When images of RNA FISH and DNA FISH are merged, HER2 mRNA foci and DNA FISH signals are closely localized, suggesting these foci are active transcription sites (HER2 mRNAs – green; HER2 DNA loci – red; nuclei – blue). The whole-field view images are presented in Supplementary Fig. S9. Bar is 5 μm.

Figure 2a shows the aggregation of HER2 nuclear exonic transcripts into discrete foci. These previously have been suggested as active transcription sites^23^. We believe that these coincide with the nuclear regions in which HER2 amplified sequences reside^24^ since our earlier studies showed that HER2 amplified sequences cluster in discrete regions of the nuclei^25^. We tested the association between HER2 mRNA foci and HER2 copy number by performing mRNA FISH and DNA FISH on the same cells. When images RNA FISH and DNA FISH are merged, aggregation of HER2 nuclear transcripts were colocalized with most HER2 DNA loci (Fig. 2c). These studies confirm that the aggregated mRNA hybridization sites occur at sites of HER2 amplification. However, mRNA signals were not associated with every region of amplified HER2 DNA suggesting that not all HER2 genes are active for transcription in steady state. We compared the levels of HER2 measured using immunoFISH with HER2 copy number measured using comparative genomic hybridization analysis in 10 different breast cancer cell lines (Supplementary Fig. S4). The correlation was high (*r* = 0.99, *p* = 4.3 × 10^−8^), suggesting that HER2 amplification status can be analyzed via immunoFISH as an alternative to DNA FISH.

Figure 2a,b shows that immunoFISH with probes targeting exonic and intronic HER2 RNA (Fig. 2a) provides information about spatial aspects of mRNA processing^16^. Specifically, it shows that the intron containing transcripts are only found in the nucleus as expected since mRNA is synthesized as a precursor mRNA (pre-mRNA) and introns are removed prior to nuclear export^26,27^. The lamin A/C staining precisely defines the nuclear envelope shape including protrusions, inclusions, wrinkles and micronuclei so that the nuclear organization of mRNA transcripts can be assessed. Micronuclei typically contain chromosome fragments that are not incorporated into daughter nuclei during cell division^28,29^ (Fig. 2b arrows) including double minute chromosomes carrying amplified sequences. Figure 2b shows the presence of unprocessed HER2 mRNA transcripts with both introns and exons indicating that at least some HER2 sequences are located in micronuclei and actively transcribed there.

### Analysis of pAKT protein, HER2 mRNA, and AKT1 mRNA during drug treatment

**Figure 3:**
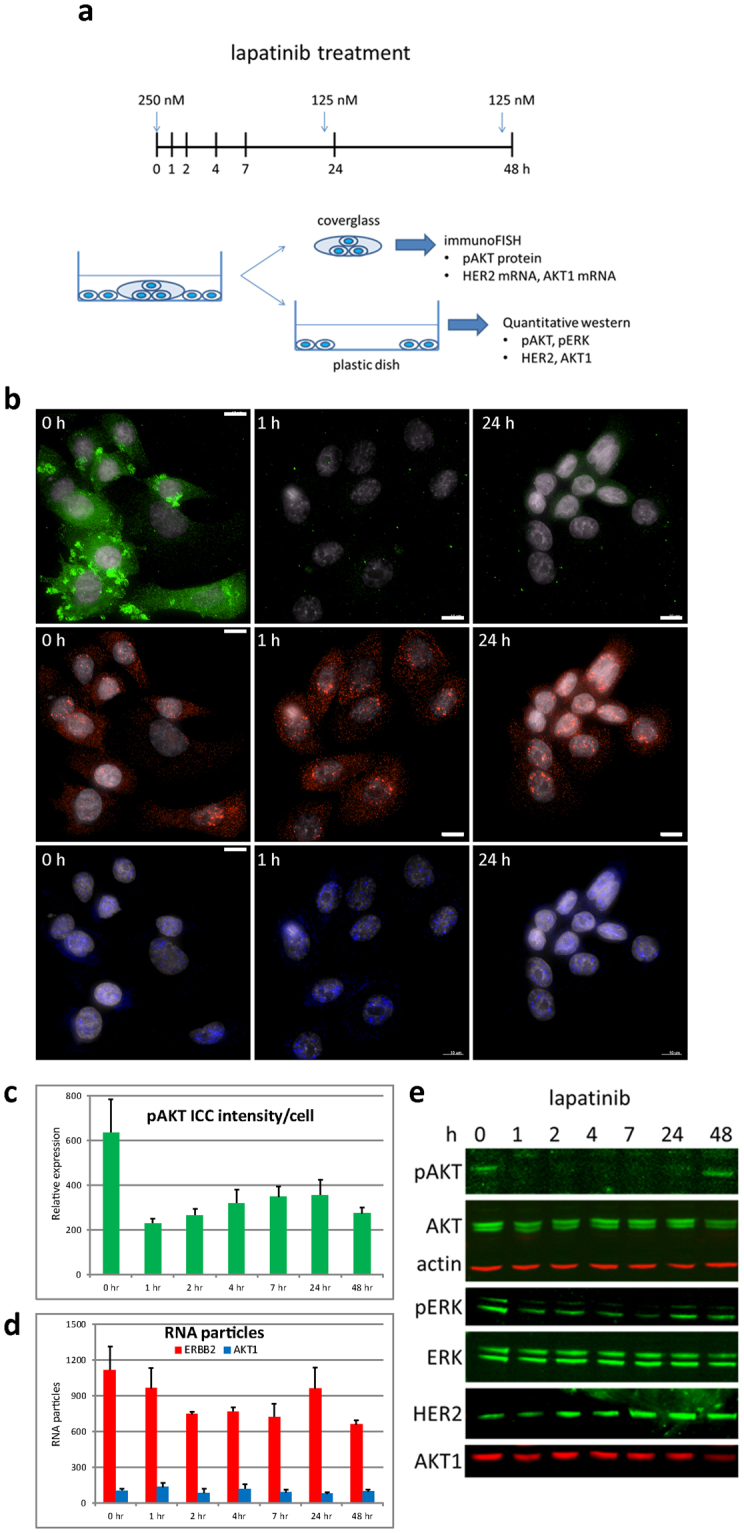
ImmunoFISH to follow *in situ* changes in HER2 and AKT1 mRNA expression, and phosphoAKT protein levels in cells treated with the HER-family tyrosine kinase inhibitor lapatinib. (**a**) Experimental design of *in situ* imaging of pAKT proteins, HER2 mRNA, and AKT1 mRNA in SKBR3 cells treated with lapatinib over a 48-h period. (**b**) Quantitative co-imaging of pAKT proteins, HER2 mRNAs, and AKT1 mRNAs via immunoFISH (pAKT protein – green; HER2mRNA – red; and AKT1 mRNA – blue; DAPI - gray) on different time points after lapatinib treated SKBR3 cells. The images of all time points are presented in Supplementary Fig. S10. (**c**) pAKT intensities in each cell of immunoFISH were measured by mean of pixel intensities. pAKT immunostaining signals were not recovered in cells grown on glass coverslips by 48 h after lapatinib treatment. (**d**) RNA particles of HER2 and AKT1 were counted in single cell level from acquired high resolution images. (**e**) Western blot analysis of lysates from SKBR3 cells grown plastic dish treated with lapatinib over a 48-h period, indicating that AKT and MAPK signaling activities were recovered through 48 h after lapatinib treatment. Western analyses of all target proteins were obtained by probing, stripping, and reprobing with each specific antibody on the same western blot membrane. Each cropped blot is divided by white space and full-length blots are presented in Supplementary Fig. S11. Bar is 10 μm.

Regulation of receptor mediated signaling typically involves coupled proteomic and transcriptional processes that influence the receptors themselves and downstream signaling pathways. Amplification of HER2, for example, activates downstream AKT signaling leading to increased proliferation and decreased apoptosis^30^. Lapatinib and similar small molecule inhibitors acts to inhibit AKT signaling^31^ but the inhibition typically is transient and full signaling is restored within ~ 48 h^32,33^. The mechanism by which AKT signaling recovers is not fully understood but typically involves both transcriptional and protein mediated processes. ImmunoFISH is well suited to analysis of these coupled processes. We illustrate that here through immunoFISH analysis of HER2 and AKT1 transcripts and phosphoAKT (pAKT) levels in SKBR3 cells at 1, 2, 4, 7, 24, and 48 h after treatment with lapatinib (Fig. 3a). HER2 and AKT1 transcripts were detected with RNA probes labeled with CAL Fluor Red 610 and Quasar670, respectively and pAKT was detected using anti-pAKT antibody/2′ antibody labeled with Alexa-488.

Figure 3b shows that there was a sharp decrease in pAKT signals of SKBR3 cells cultured on glass coverslips within 1 h of receiving lapatinib treatment and that pAKT levels recovered to ~ 50% of pretreatment levels within 24 h after treatment (Fig. 3c). By 7 h after lapatinib treatment, HER2 mRNA particles decreased to the 65%, but recovered to the 86% at 24 h after treatment (Fig. 3d). When we performed western analysis of cell lysates cultured on plastic surface, pAKT levels were down-regulated following lapatinib treatment, but they fully recovered by 48 h after treatment, as previously is concordant with an increase in HER2 proteins at 24 h, detected in the western blot analysis (Fig. 3e), following the recovery of the HER2 RNA level at 24 h (Fig. 3d). The level of AKT1 RNA particles and AKT1 proteins fluctuated throughout the 48 h after lapatinib treatment (Fig. 3d,e).

**Figure 4:**
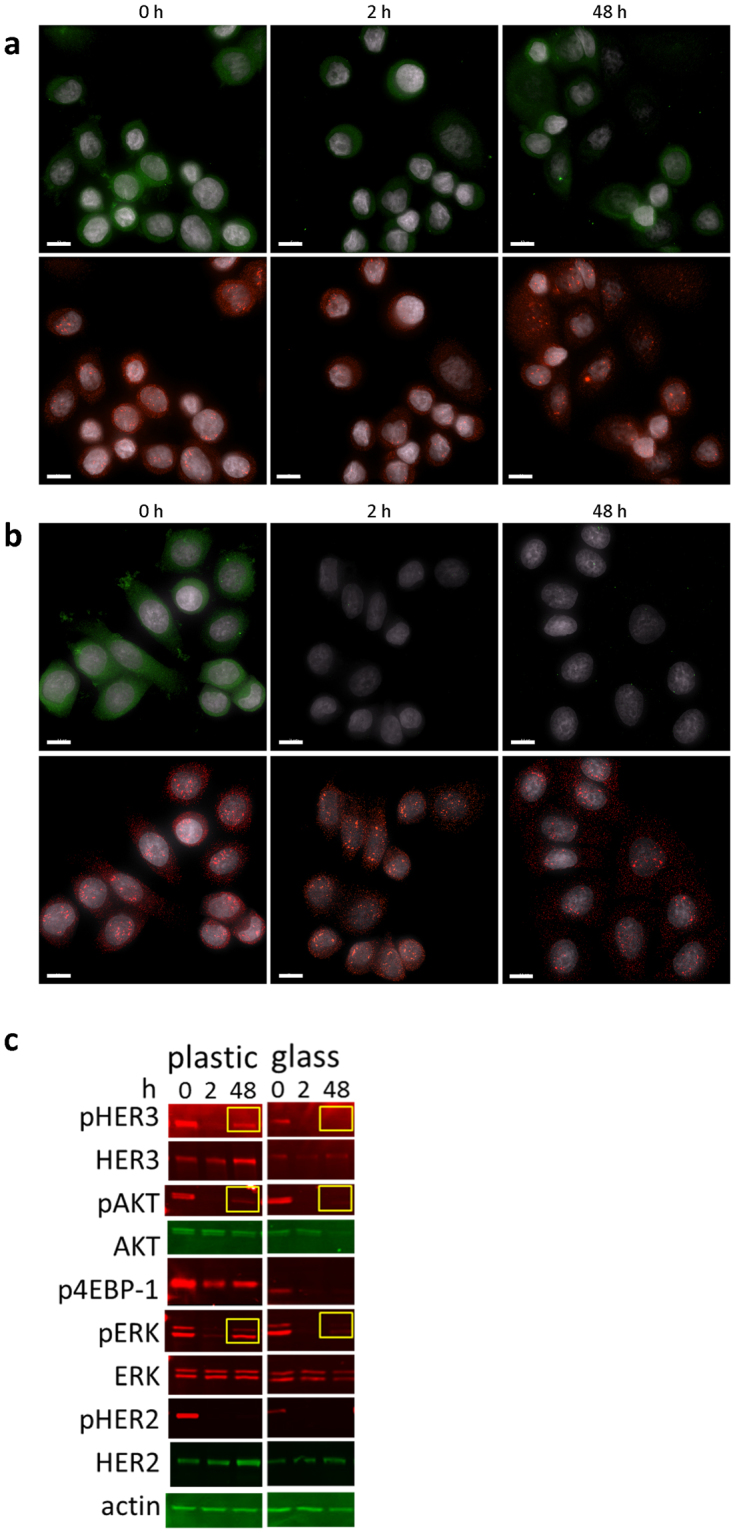
Comparison of pathway activation in SKBR3 cells treated with lapatinib grown on plastic coverslips and on glass coverslips. (**a**) ImmunoFISH of cells on plastic coverslips (pAKT protein – green; HER2 mRNA – red; and DAPI - gray). pAKT was decreased on 2 h after lapatinib treatment but recovered on 48 h. (**b**) ImmunoFISH of cells on glass coverslips. pAKT was not recovered on 48 h after lapatinib treatment. (**c**) Western blot analysis. pHER3, pAKT, and pERK were recovered in SKBR3 cells grown on plastic coverslips treated with lapatinib, but not in SKBR3 cells grown on glass coverslips and treated with lapatinib. Western analyses of all target proteins were obtained by probing, stripping, and reprobing with each specific antibody on the same western blot membrane for plastic and glass, respectively. Each cropped blot is divided by white space and full-length blots are presented in Supplementary Fig. S12. Bar is 10 μm.

We noted an interesting difference in pAKT levels following treatment with lapatinib in SKBR3 cells grown on plastic surfaces and glass coverslips. Figure 4a,b show immunoFISH analyses of pAKT levels at 0, 2 and 48 h suggesting that the pAKT levels in the cells grown on plastic coverslips had recovered to pretreatment levels by 48 h but cells grown on glass coverslips had not (Figs 3, 4c). Western blot analyses support this observation and further show that the recovery of pHER3, pAKT, and pERK levels differs for cells grown on the plastic coverslips and glass coverslips (Fig. 4c).

### ImmunoFISH analysis formal-fixed, paraffin embedded (FFPE) tissues reveals the molecular architecture of HER2+ breast cancers

**Figure 5:**
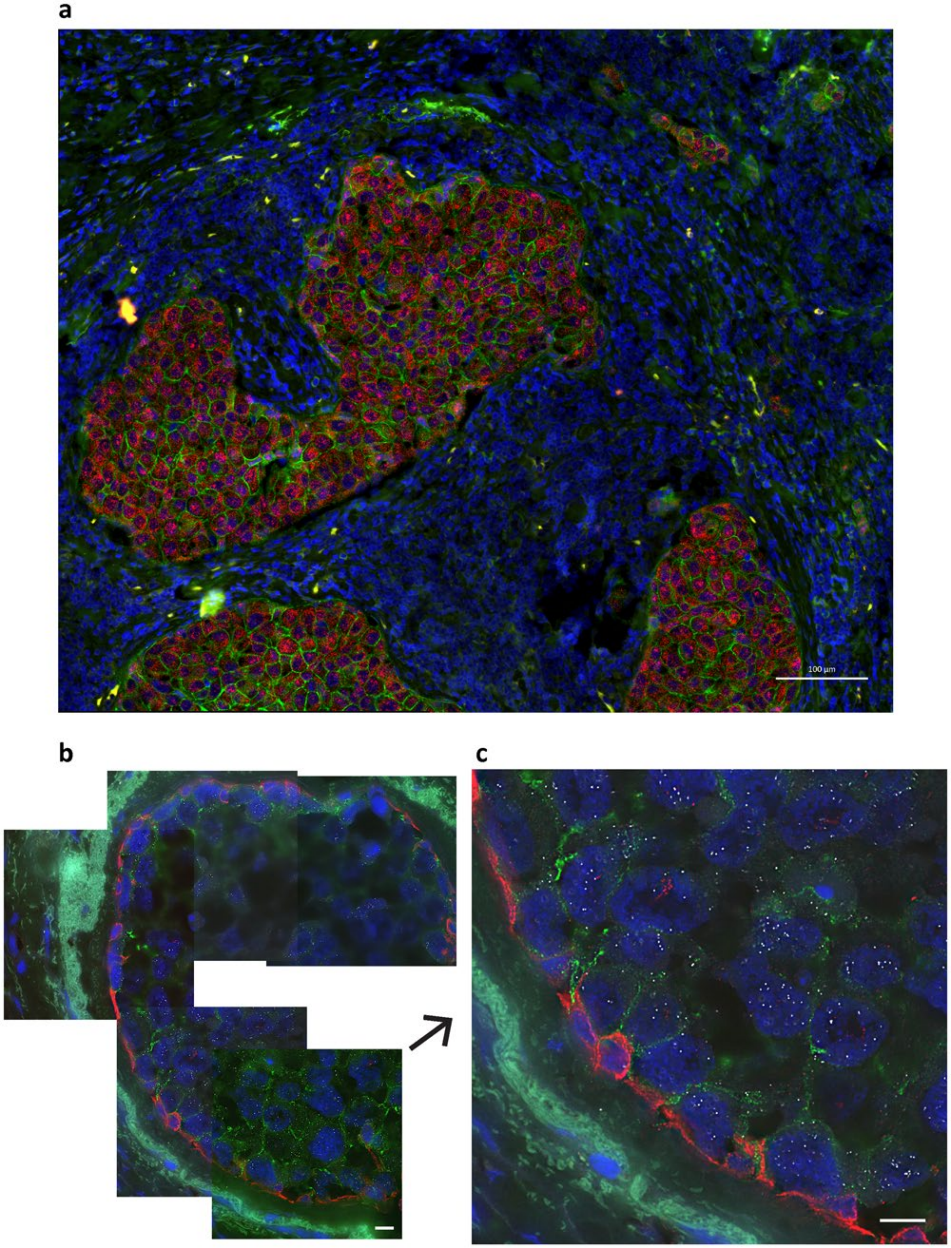
ImmunoFISH to analyze protein levels and mRNA expression in human formalin-fixed, paraffin-embedded (FFPE) tissue sections. Please note HER2 RNAs were detected by bDNA-probe FISH, followed by immunostaining of HER2 or HER2/KRT14 proteins. (**a**) Overview of HER2 protein (green) and HER2 mRNA (red) in a DAPI stained (blue) HER2+ invasive breast cancer. Detection of both HER2 protein and mRNA is apparent and there is a remarkable interface separating the HER2+ tumor from surrounding microenvironment. Nine tile images (3 × 3) using a Zeiss Axio Imager.M2 with a Plan-Apochromat 20X, NA = 0.8 objective, were obtained, stitched and fused to make single image. Bar is 100 μm. (**b**) High resolution co-imaging of HER2 protein (green), HER2 mRNA (white) and the myoepithelial protein, KRT14 (red) on HER2 + breast tumor nest. Five images around a tumor nest were obtained using a Deltavision Automated Widefield microscopy (60X objective, NA = 1.42). The KRT14 positive cells give clear outlines of the tumor nest. Bar is 10 μm. (**c**) Zoomed image shows that the KRT14 positive cells do not express HER2 mRNA confirming that they are not HER2+ derived tumor cells but likely normal myoepithelial cells. Bar is 10 μm.

We applied immunoFISH to analysis of HER2 protein levels and HER2 mRNA expression in human tumor FFPE tissue sections. We accomplished this by applying immunoFISH to FFPE tissue sections that were first deparaffinized and then pressure cooked for antigen retrieval. We acquired 9 tile images (3 × 3) using a Zeiss Axio Imager.M2 with a Plan-Apochromat 20X, NA = 0.8 objective, and fused them to make a single image using ZEN software (Zeiss) (Fig. 5a). Figure 5a shows an analysis of HER2 protein and HER2 mRNA in a DAPI stained, HER2+ invasive breast cancer. Detection of both protein and mRNA is apparent. Equally apparent is the existence of the remarkable interface separating the HER2+ tumor cells from surrounding stromal and immune cells. This interface has been reported as comprised of a layer of normal myoepithelial cells that surround and contain early cancer cells by secreting basement membrane proteins such as fibronectin, collagen IV, nidogen and laminins^34^. This isolating barrier is thought to be lost during cancer invasion^35^. We investigated the idea that the barrier is formed by normal myoepithelial cells (as opposed to transdifferentiated tumor cells) by staining for HER2 protein, HER2 mRNA and the myoepithelial protein, KRT14. Figure 5b,c show the KRT14 positive cells that line the tumor nest. Importantly, the high resolution image in Fig. 5c shows that the KRT14 positive cells do not express elevated levels of HER2 mRNA confirming that they are normal myoepithelial cells. We attribute the differences in HER mRNA levels and granule size between Fig. 5a,b and c to the fact that HER2 amplification and expression were substantially higher in the tumor analyzed in Fig. 5a than that analyzed in Fig. 5b,c. In addition, Fig. 5a was acquired using a 20X/0.8NA objective while Fig. 5b,c were acquired using a higher resolution 60X/1.42NA objective. Thus, Fig. 5b,c show more single HER2 RNA particles than Fig. 5a because they were fewer in number and imaged at higher resolution. Additional differences may be due to differences in fixation between these two clinical samples.

## Discussion

We have developed “immunoFISH” to quantify proteins and mRNA simultaneously in a spatially defined manner and demonstrated the utility of the approach in several studies including: (a) Analysis of the impact of lapatinib on the levels and spatial organization of HER2 and AKT1 mRNAs and proteins in SKBR3 cells. These studies revealed overall changes but also show substantial heterogeneity between cells that may contribute to resistance. Measures of heterogeneity between cells may guide the development of therapeutic strategies that are effective against heterogeneous populations. An interesting byproduct of this study was the discovery that HER2 signaling through AKT is different in cells grown on plastic and glass. This is important since results from imaging studies performed on cells grown on glass (because of its superior optical quality) may not be biologically comparable to studies conducted using cells grown in plastic multiwell plates. (b) Elucidation of the nature of the myoepithelial barrier that separates tumor from stromal cells in HER2+ breast cancer. Our studies clearly showed that the myoepithelial cells at the border between the tumor cells and stromal cells do not express elevated levels of HER2 protein or mRNA and so they are normal myoepithelial cells that are recruited to the tumor and/or remain as part of the normal myoepithelium that surrounded the duct in which the tumor arose or into which it migrated. This raises the possibility of manipulating the normal myoepithelial layer to increase immune cell access to tumor cells in which lesions where exclusion occurs. (c) Analysis of the spatial organization and composition of HER2 mRNA transcripts. Specific findings include co-localization of RNA aggregates and amplified HER2 DNA loci in interphase nuclei, the presence of unprocessed mRNA transcripts in micronuclei and the expected loss of intronic sequences from HER2 mRNA transcripts as they translocate from the nucleus to the cytoplasm.

Overall, the immunoFISH procedures reported here generally are robust. However, we have encountered technical issues in some situations that should be noted. For example, we found that the mRNA signal-to-noise ratio was lower in FFPE tissues than in cultured cells due to intrinsic background autofluorescence. We overcame this by using branched DNA (bDNA) probes to amplify the mRNA signals^36^. We also found that the efficiency of immunofluorescence staining was significantly reduced by the conditions needed for stringent bDNA-probe FISH. We overcame this by performing RNA FISH first followed by immunofluorescence staining. Finally, we found in sequential analyses of HER2 mRNA and DNA, that the nuclei shrank during DNA FISH possibly due to pepsin treatment and high temperature incubation needed for DNA FISH. We corrected for this by rescaling nuclei from DNA FISH aligning to those from RNA FISH. After this correction, aggregates of HER2 RNA particles in nuclei co-localized with most of the of the amplified HER2 DNA signals. We also found that while simultaneous addition of RNA probes and primary antibodies against protein targets worked well in most situations, some situations (e.g. in FFPE) staining could be improved by performing FISH first followed by immunostaining for protein as recommended by Kochan *et al*.^37^.

## Methods

### Cell culture and drug treatments

The breast cancer cell lines were grown, following Neve *et al*.^20^. To inhibit HER2 tyrosine kinase, SKBR3 cells were cultured in McCoy’s 5A medium (Life Technologies) in the presence of 10% fetal bovine serum for 48 h with 250 nM lapatinib, and an additional 125 nM of lapatinib was added in the 23^rd^ and 47^th^ h time points to supplement possibly degraded lapatinib.

### RNA-seq data

Raw read counts of RNA-seq data for HER2, GAPDH, and beta-actin were acquired from the previously published data bank of breast cancer cell lines^21^.

### FFPE tissue sections

De-identified human breast cancer FFPE tissue sections were obtained from Oregon Health and Science University (OHSU) Knight BioLibrary (Portland, OR). The tissue sample analysis was reviewed as STUDY00016928 by the OHSU IRB on March 23, 2017 and judged as “not research involving human subjects”. All analysis methods were performed in accordance with the guidelines summarized in the OHSU Institutional Biosafety Committee document, IBC-11-11, approved June 24, 2017. HER2 TMA with 10 cases/10 cores of breast invasive ductal carcinoma was purchased from US Biomax Inc.

### siRNA treatment

SKBR3 cells were transfected with 20 nM siRNAs (Qiagen) of Human HER2 siRNA (Hs_ERBB2_14), Human HER2 siRNA (Hs_ERBB2_15), AllStars Negative Control siRNA, using siLentFect™ Lipid Reagent for RNAi (BIO-RAD) when they reached 50% confluence (24 h after plating). Cells were fixed in 4% formaldehyde at room temperature for 10 min 72 h after transfection for immunoFISH.

### ImmunoFISH

Cells were prepared for immunoFISH by fixing in 4% formaldehyde at room temperature for 10 min. They were then permeabilized in 70% ethanol at 4 °C for at least one h followed by prehybridization with wash buffer (2X SSC, 10% formamide) at room temperature for 5 min. Then the cells were incubated overnight at 37 °C with Stellaris FISH Probes (Biosearch Technologies) and primary antibodies in a hybridization solution (HS; 10% Dextran sulfate, 2X SSC, 10% formamide) in a dark and humid chamber. The Stellaris Probes were comprised of forty-eight 20-nucleotide probes targeting RNAs each 3′ labeled with single fluorophore^38^. The probe sets were designed using the Stellaris Probe Designer version 1.0 (http://www.singlemoleculefish.com/). After hybridization, the cells were washed twice in wash buffer for 20 min at 37 °C. The secondary antibodies conjugated with Alexa488 (Invitrogen) were then applied in HS at room temperature for 1 h, washed 2X in wash buffer for 20 min at room temperature in a dark and humid chamber. Finally, the slides were washed in 2X SSC for 5 min and mounted under coverslips in Prolong Gold with DAPI on microscopic slides. For immunoFISH on FFPE tissue sections, 5 μm sections on glass slides were baked for 1 h 60 °C, deparaffinized, then were treated for antigen retrieval. First, HER2 RNA FISH was performed on FFPE sections using RNAscope® Probe- Hs-ERBB2, based on the RNAscope Fluorescent Multiplex Kit user manual (ACD). Sections then were washed with PBS, incubated with blocking solution (10% goat serum, 1% BSA, PBS) for 30 min at RT, then primary antibodies in Ab solution (5% goat serum, 1% BSA, PBS) at 4 °C overnight. Sections were washed the next day with PBS, 0.025% Tween for 5 min three times, and secondary antibodies conjugated with Alexa488 and Alexa647 (Invitrogen) were then applied in Ab solution at room temperature for 1 h, washed 3X PBS, 0.025% Tween for 5 min three times, then mounted under coverslips in Prolong Gold with DAPI. Anti-HER2 antibody (Sigma-Aldrich 3B5), anti-lamin A/C antibody (Sigma-Aldrich SAB4200236), anti-pAKT antibody (Cell Signaling 4060), anti-Cytokeratin 14 antibody (abcam ab7800) were used for primary antibodies.

### Image acquisition

Images of 0.2 μm optical sections were acquired, using a Deltavision CoreDV Automated Widefield microscopy (60X objective, NA = 1.42) with a Photometrics Coolsnap ES2 HQ camera. The raw images were deconvoluted with SoftWoRx ™ image restoration software (GE Healthcare) to remove focus blurr or, more specifically, reassign out-of-focus light contributions to their proper location in the source image. The images were then reconstructed into 3D visualizations. The 3D images showed RNA molecules as bright “particles,” with a size of ~0.25 μm and a distinct signal from background noise. RNA particles were counted using a Spot Detection program from IMARIS software (Bitplane) as follows: the 3D images were filtered with a Mexican Hat Effect algorithm to subtract background signals. An automatic threshold was applied by calculating intensities from all spots based on statistics supplied by IMARIS. The threshold was refined manually to ensure the detection corresponds to RNA particle spots identified by eye. The number of total spots in an image was counted by IMARIS, and then the total was divided by the number of nuclei to obtain the absolute number of transcripts in single cells. HER2 protein intensities in immunoFISH level were determined by mean pixel intensities of segmented cells using QiTissue software (Quantitative Imaging Systems). Nuclei and cytoplasm were automatically segmented with a model driven algorithm, and interactively checked and corrected for proper cell boundaries.

### Western blot analysis

Cells were lysed in a RIPA buffer (150 mM NaCl, 1.0% IGEPAL® CA-630, 0.5% sodium deoxycholate, 0.1% SDS, 50 mM Tris, pH 8.0.) with Halt Protease and Phosphatase Inhibitor Cocktail (Pierce) and then cleared by centrifugation. Protein concentration was estimated with a BCA assay (Pierce). Proteins present in cell lysates (30 μg) were resolved by SDS-PAGE and transferred onto polyvinylidene difluoride (PVDF) membrane. Membranes were stained with Ponceau S to confirm equal loading and probed, stripped with NewBlot™ PVDF Stripping Buffer (LI-COR), and reprobed with antibodies specific for phospho-AKT (Cell Signaling), AKT(Cell Signaling), phosphor-ERK (Cell Signaling), ERK (Cell Signaling), phospho-HER2 (Cell Signaling), HER2 (Sigma-Aldrich), phospho-HER3 (Cell Signaling), HER3 (Cell Signaling), AKT1(Cell Signaling), phosphor-4EBP-1(Cell Signaling) or actin (Santa Cruz). Immunoreactive proteins were detected and quantified using infrared fluorescence, IRDye® secondary antibodies, and Odyssey® imagers (LI-COR).

### HER2 RNA-DNA FISH

SKBR3 cells were cultured on 8 well, Lab-Tek Chamber Slide (Thermo Scientific Nunc), fixed in 4% formaldehyde at room temperature for 10 min. Cells were permeabilized in 70% ethanol at 4 °C for at least one h and then were prehybridized with wash buffer (2X SSC, 10% formamide) at room temperature for 5 min. Then the cells were incubated with HER2 Stellaris FISH Probes in a hybridization solution (10% Dextran sulfate, 2X SSC, 10% formamide) overnight at 37 °C. Following two washes with a wash buffer for 30 min at 37 °C, the coverslips were mounted in SlowFade Gold on microscopic slides. DAPI was included in the second wash buffer for nuclear counterstaining. RNA FISH signals were imaged using Zeiss ApoTome2 on AxioImager (63X objective, NA = 1.4). To remove RNA FISH signals, cells were incubated with PBS/0.05% Triton X-100 3X for 10 min, treated with RNase A (25 μg/ml) for 30 min at 37 °C, then washed with PBS. No detectable RNA FISH signals were confirmed by Zeiss ApoTome2. For consecutive DNA FISH, cells were washed with 2X SSC at RT, incubated with CytoZyme Stabilized Pepsin (SciGene) for 5 min at 37 °C, then dehydrated with 70%, 85%, 100% serial ethanol treatment. Then the cells were incubated with ERBB2 Orange + Copy Control 17 Green DNA FISH Probe (BioCare Medical) at 85 °C for 2 min then at 39 °C overnight. Cells were washed with 0.4X SSC/0.3% NP-40 for 2 min at 72 °C, 2X SSC/0.3% NP-40 for 2 min at RT, then DAPI/2X SSC for 10 min at room temperature. DNA FISH signals on same cells imaged for RNA FISH were imaged using Zeiss ApoTome2 on AxioImager (63X objective, NA = 1.4). Images from the multiple image cycles were reconstructed using QiTissue software, to automatically register the location of cells and nuclei, and to perform warping to correct for nuclear shrinkage and deformation introduced by the DNA FISH labeling procedure. To do so, control points were detected on the nuclear boundaries and each nucleus was individually corrected by piecewise bicubic interpolation. The resulting images show proper overlay of RNA and DNA signals in aligned nuclei, combined with the unaltered HER2 signals in the cytoplasm.

### Comparative genomic hybridization (CGH)

Genomic DNA of each cell line was analyzed using Array CGH with the latest version of OncoBAC arrays for DNA copy. The OncoBAC arrays were comprised of 2402 P1, PAC, or BAC clones^39^. About 80% of the clones on the OncoBAC arrays contained genes and STSs implicated in cancer development or progression. All clones were printed in quadruplicate. DNA samples for array CGH were labeled generally as described previously^40–42^. Briefly, we random-prime labeled 500 ng of test (cell line) and reference (normal female, Promega) genomic DNA with CY3-dUTP and CY5-dUTP (GE Healthcare Life Science), respectively, using Bioprime kit (Invitrogen). Labeled DNA samples were coprecipitated with 50 mg of human Cot-1 DNA (Invitrogen), denatured, hybridized to BAC arrays for 48–72 h, washed, and counterstained with DAPI. Data processing Array CGH data image analyses were performed as described previously^43^. Array probes missing in more than 50% of samples in the OncoBAC or scanning array data sets were excluded in the corresponding set. Array probes representing the same DNA sequence were averaged within each data set.

### Data availability

The datasets generated during and/or analysed during the current study are available from the corresponding author on reasonable request.

## Electronic supplementary material

Supplementary Figures
