# Supplementary material for: Quantitative, in situ analysis of mRNAs and proteins with subcellular resolution
Source: Sci Rep. 2017 Nov 28;7:16459. doi: 10.1038/s41598-017-16492-1 (PMC5705767; doi:10.1038/s41598-017-16492-1)

Supplementary information

Quantitative, in situ analysis of mRNAs and proteins with subcellular resolution

Sunjong Kwon, Koei Chin, Michel Nederlof, and Joe W. Gray

a

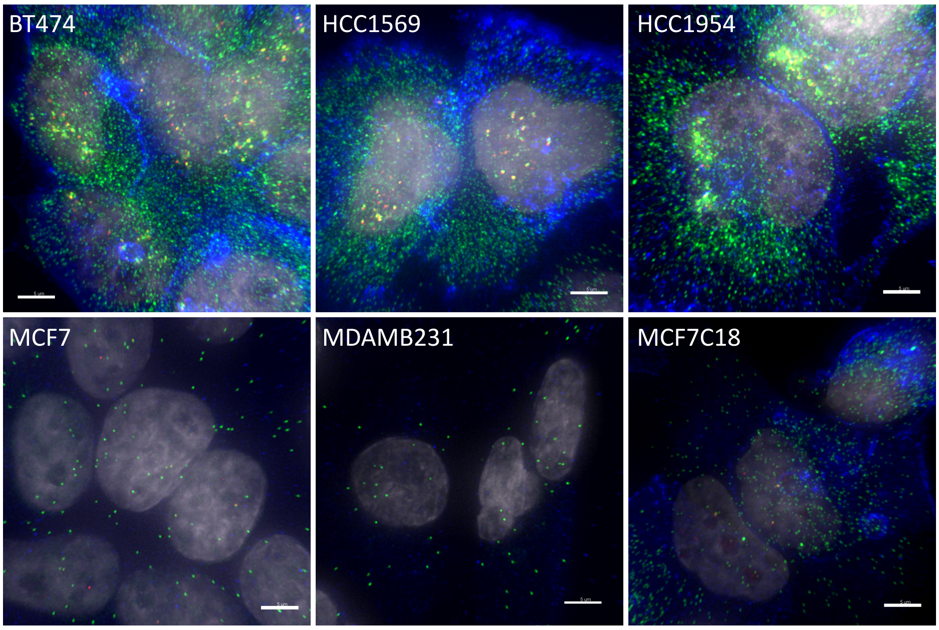

b

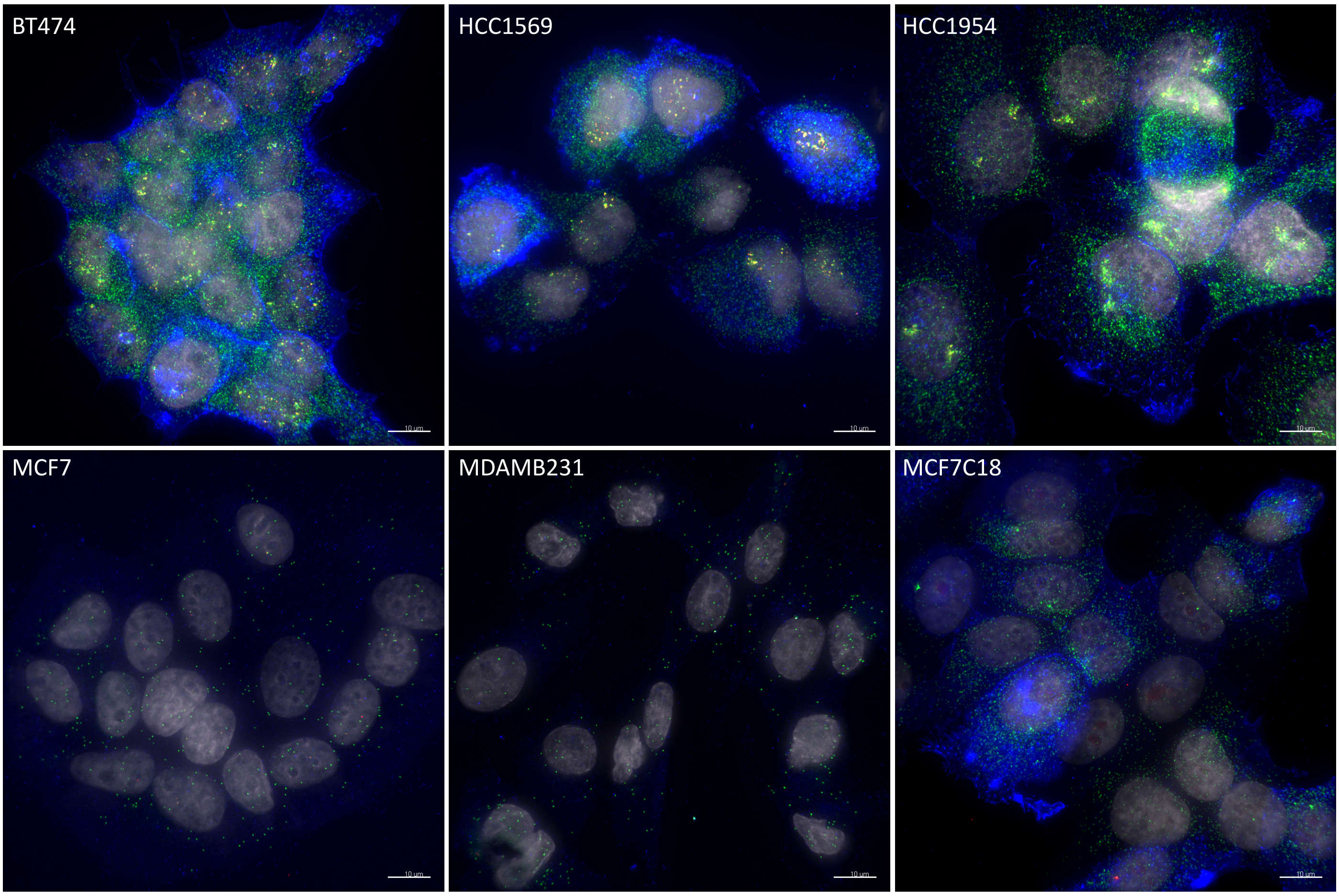

Supplementary Fig S1. (**a**) Simultaneous co-imaging of HER2 proteins (blue), HER2 mRNAs (green), intronic RNAs (red), and nuclei (gray) of different breast cancer cell lines. Both RNA particles and proteins are highly detected in HER2 amplified cell lines BT474, HCC1954, HCC1569, and MCF7C18. Bar is 5 μm. (**b**) Whole-field view images. Bar is 10 μm.

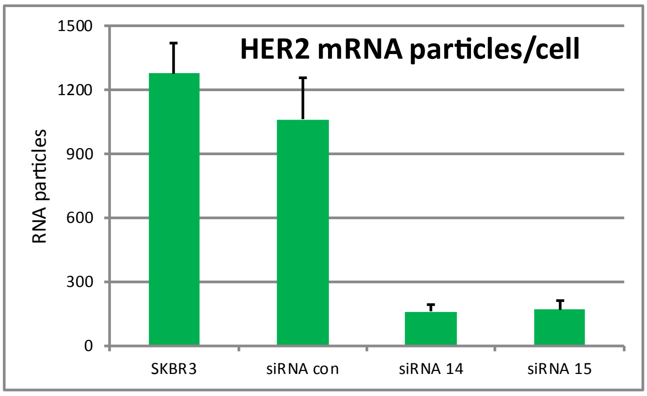

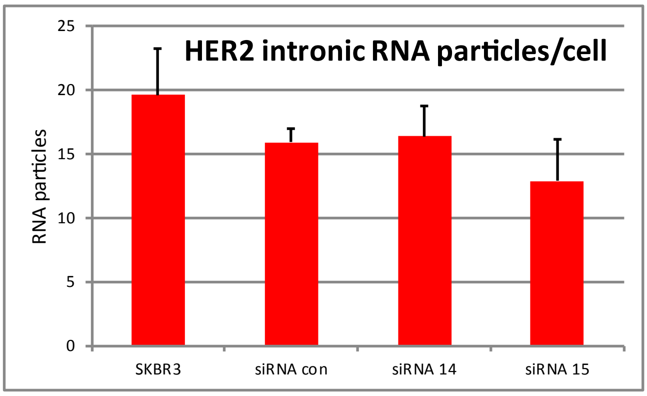

Supplementary Fig S2. RNA particles counting of HER2 mRNAs and intronic RNAs on SKBR3 with HER2 knock-down by siRNAs. RNA signals to target HER2 intron sequences, which were exclusively detected in the nuclei, were not clearly affected by siRNA treatment.

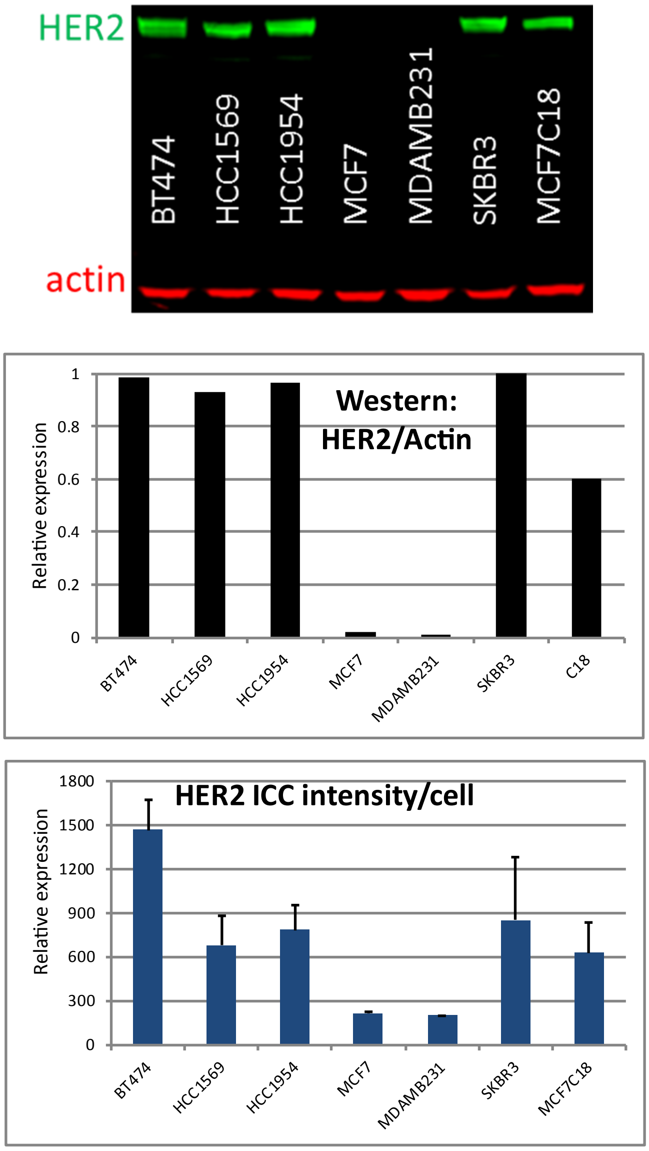

Supplementary Fig S3. Comparison of HER2 protein levels by western blotting and immunoFISH of different breast cancer cell lines.

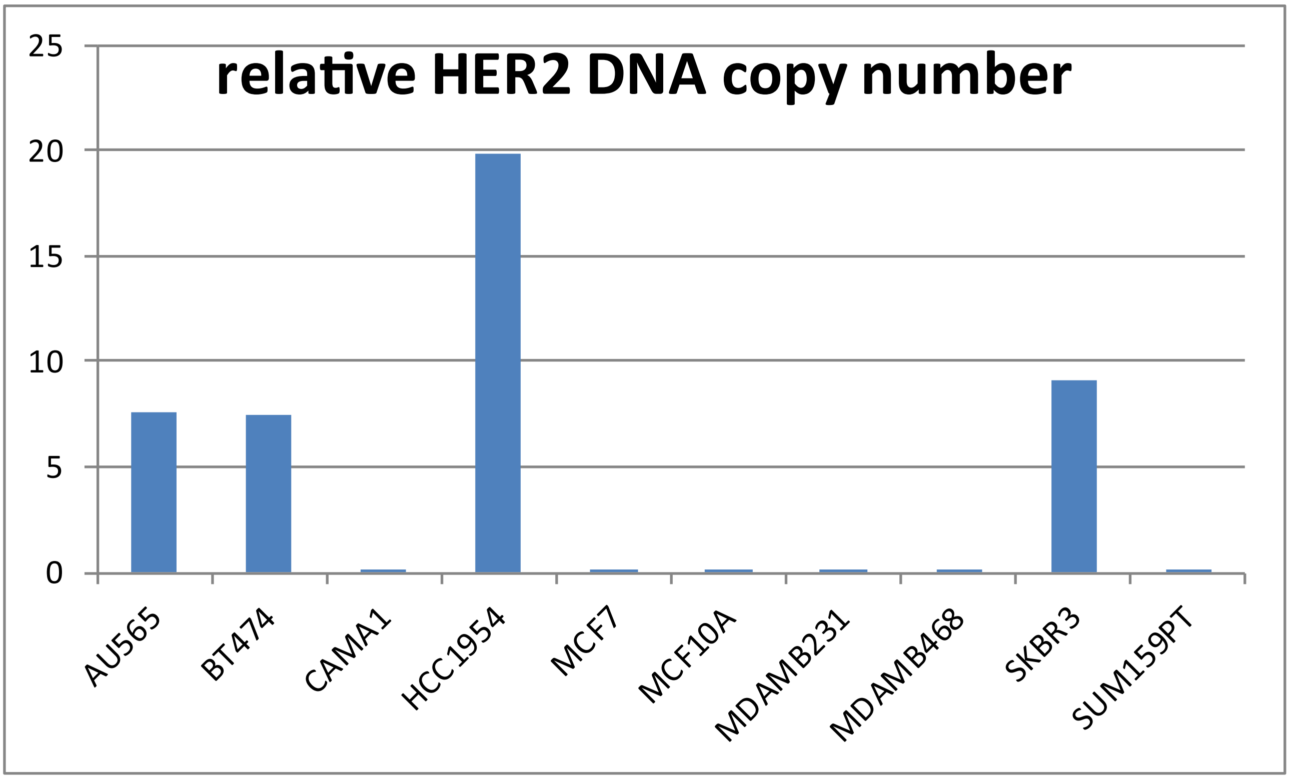

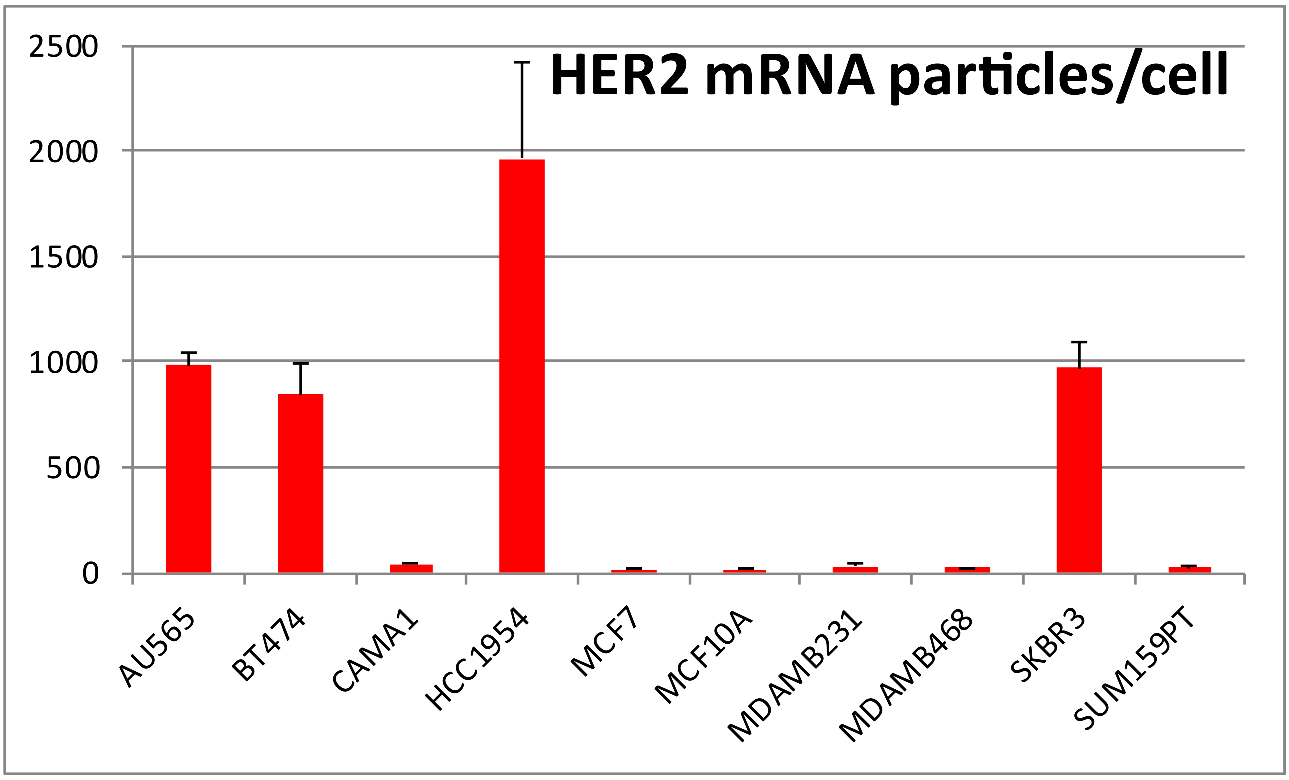

Supplementary Fig S4. The numbers of HER2 RNA particles in immunoFISH are closely related with DNA copy number from comparative genomic hybridization data.

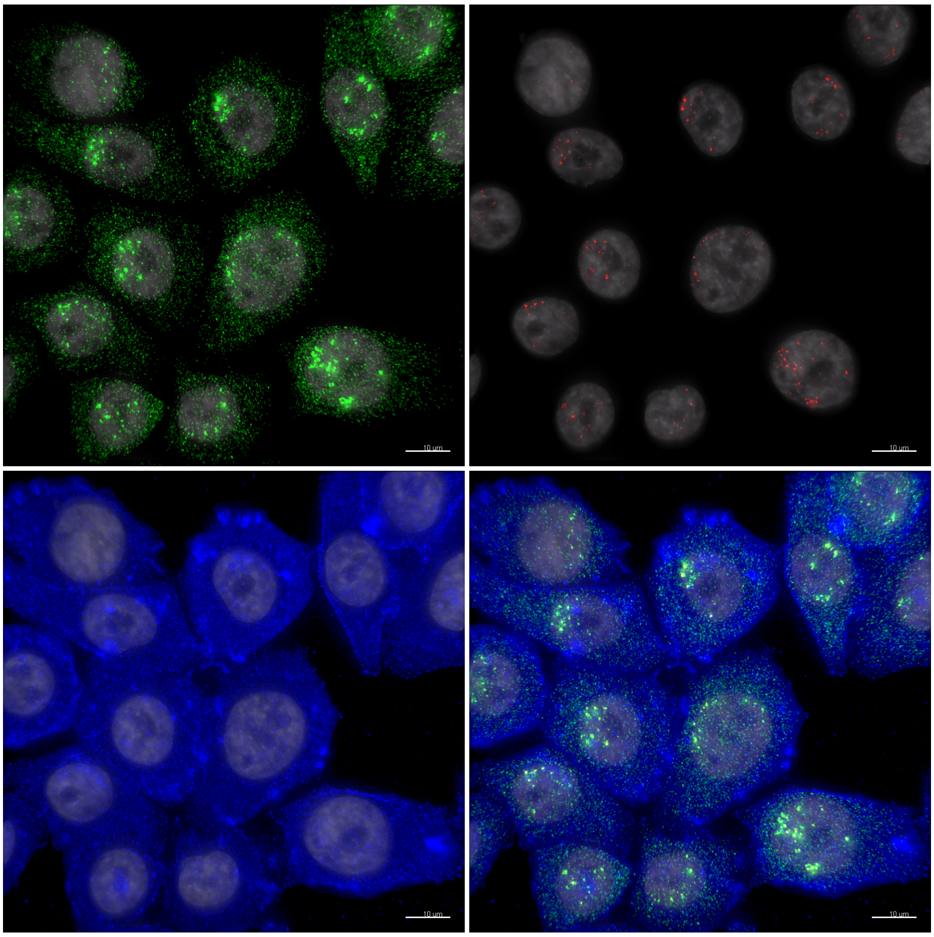

Supplementary Fig S5. Whole-field view images of Fig 1a. Bar is 10 μm.

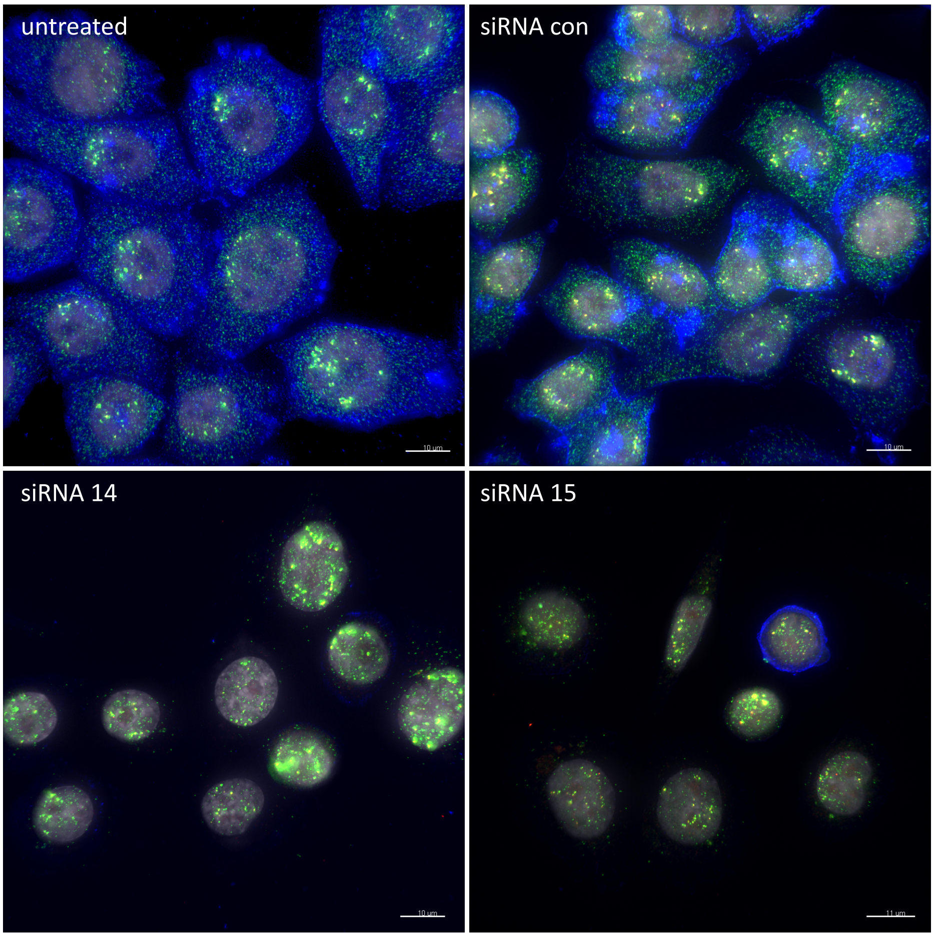

Supplementary Fig S6. Whole-field view images of Fig 1b. Bar is 10 μm.

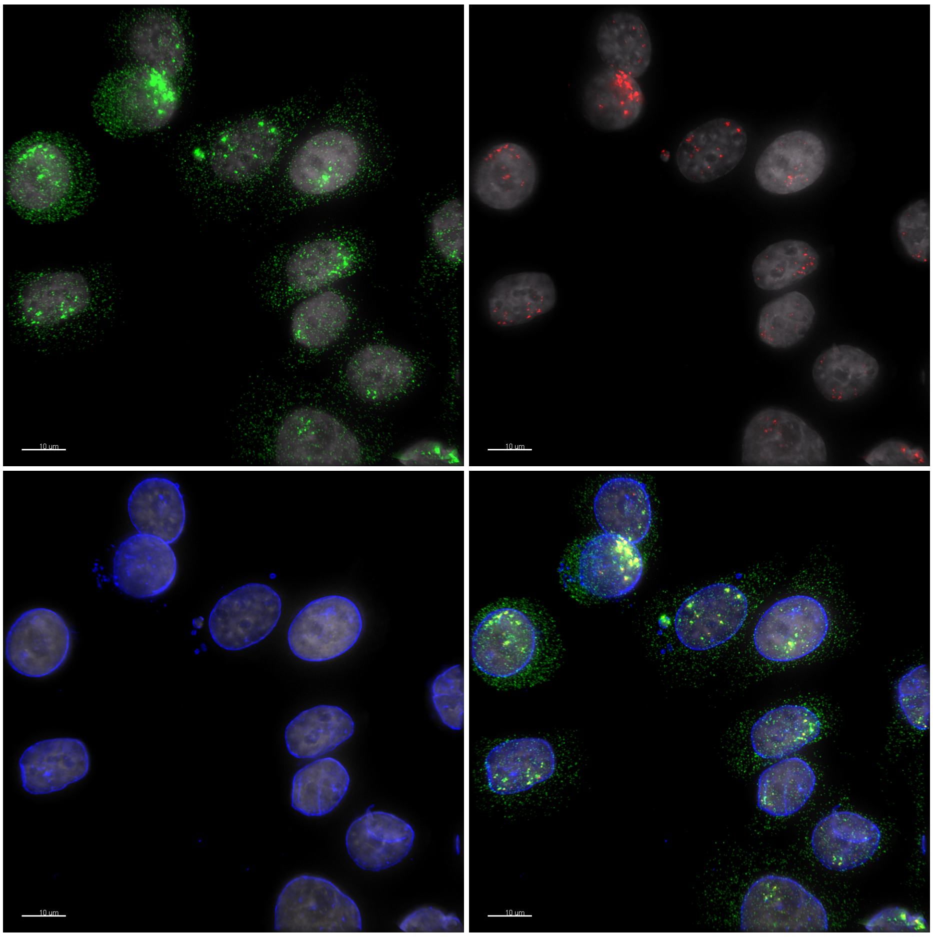

Supplementary Fig S7. Whole-field view images of Fig 2a. Bar is 10 μm.

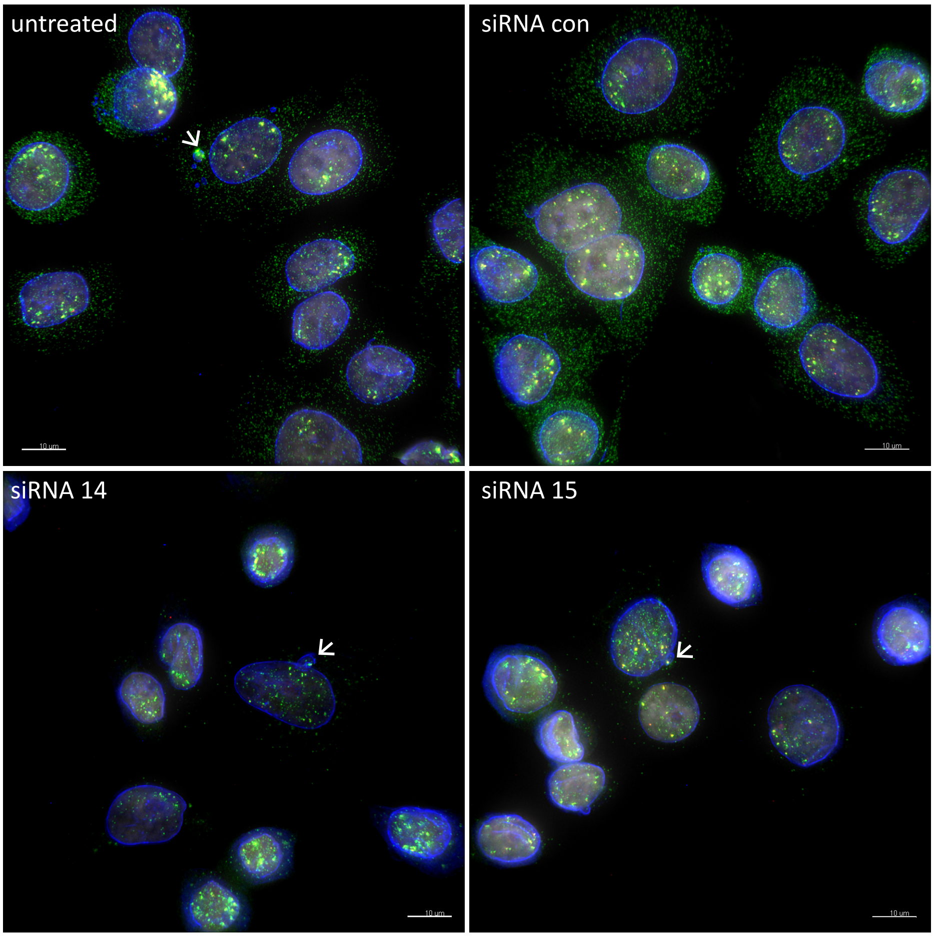

Supplementary Fig S8. Whole-field view images of Fig 2b. Bar is 10 μm.

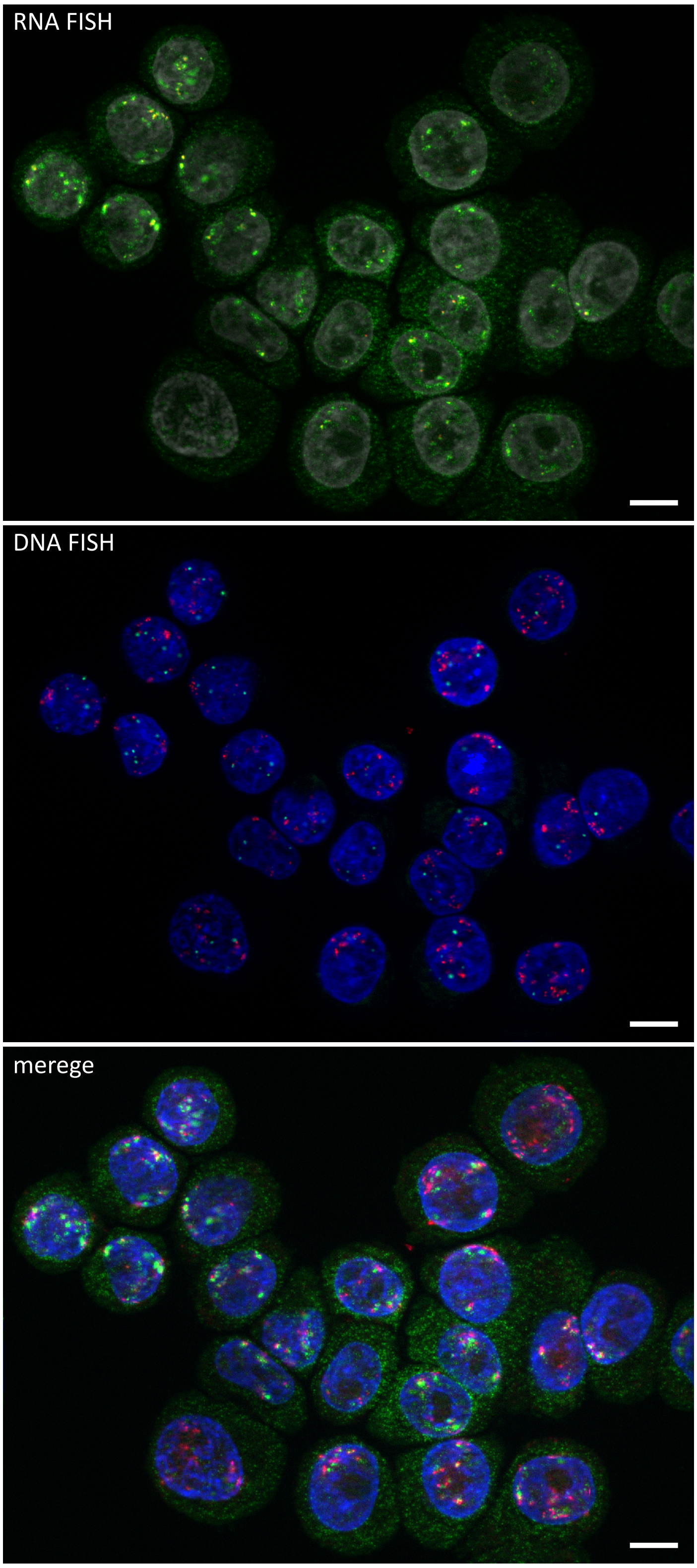

Supplementary Fig S9. Whole-field view images of Fig 2c. Bar is 10 μm.

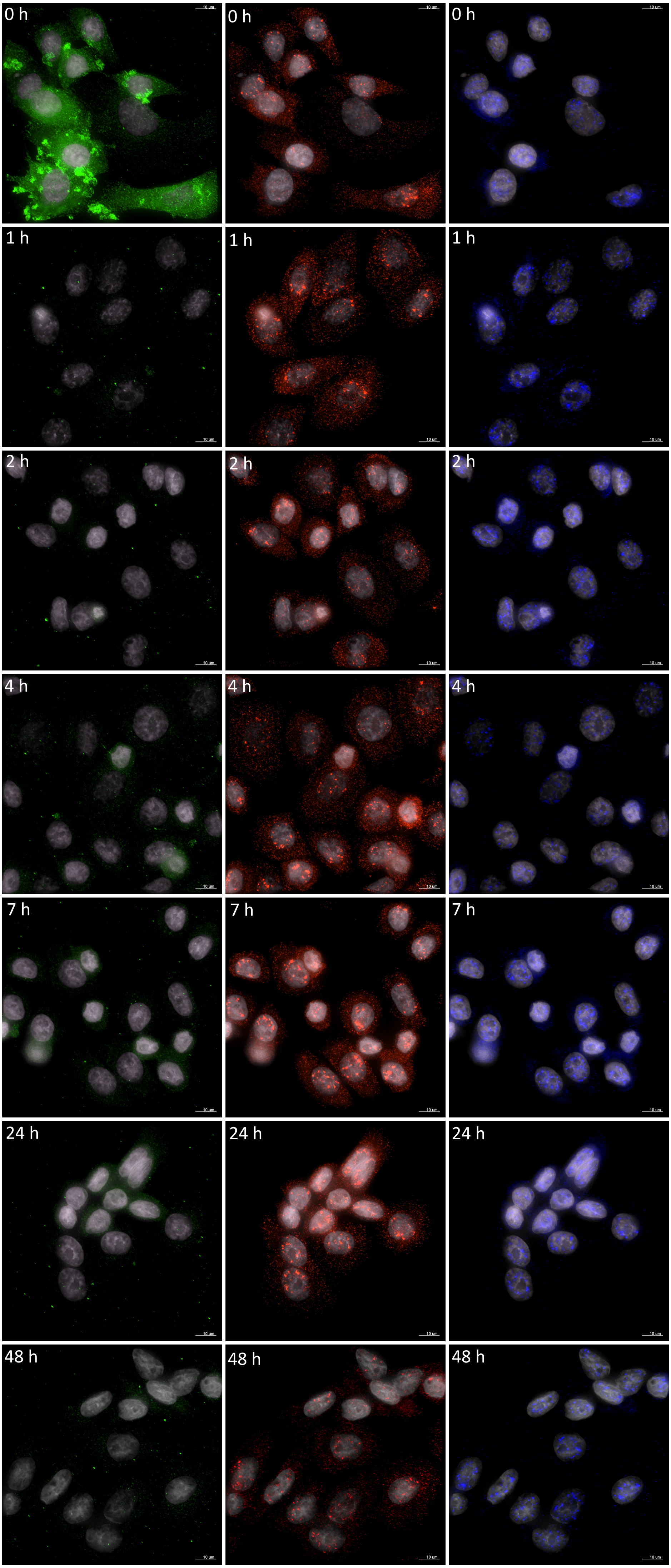

Supplementary Fig S10. Images of all time points of pAKT proteins (green), HER2 mRNAs (red), and AKT1 mRNAs (blue) of Fig 3b. Bar is 10 μm.

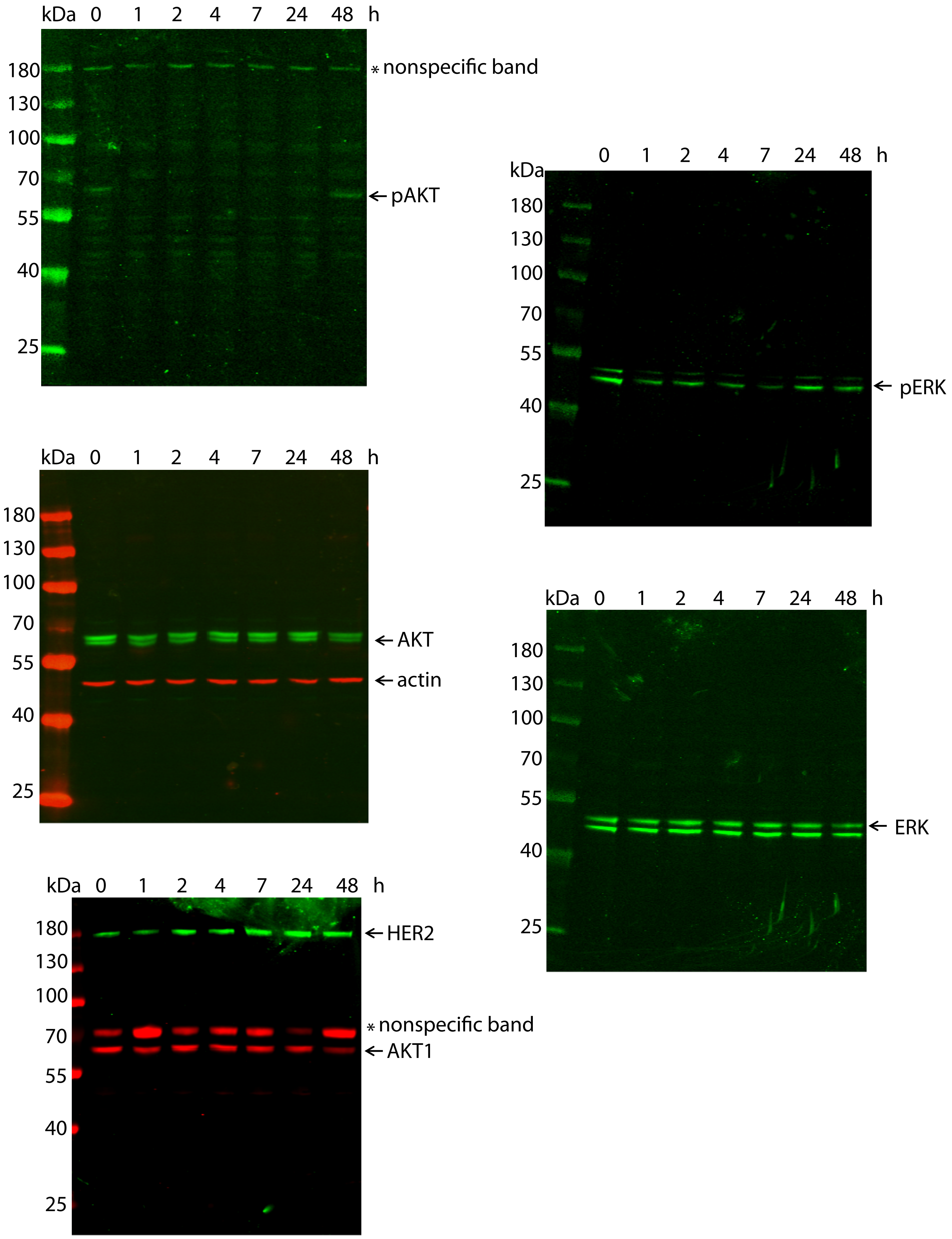

Supplementary Fig S11. Full-length western blot images of Fig 3e.

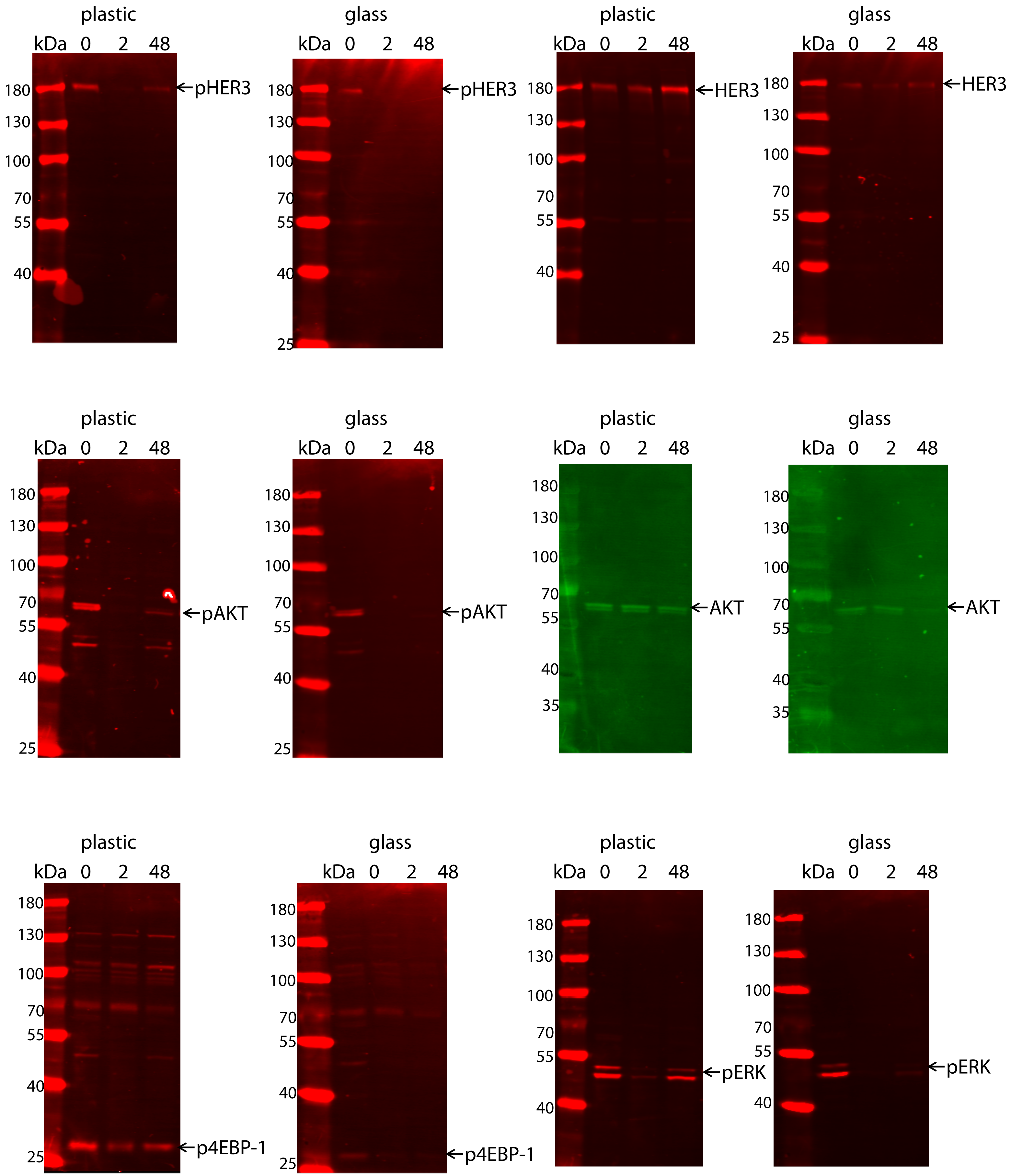

Supplementary Fig S12. Full-length western blot images of Fig 4c.

Supplementary Fig S12. continued

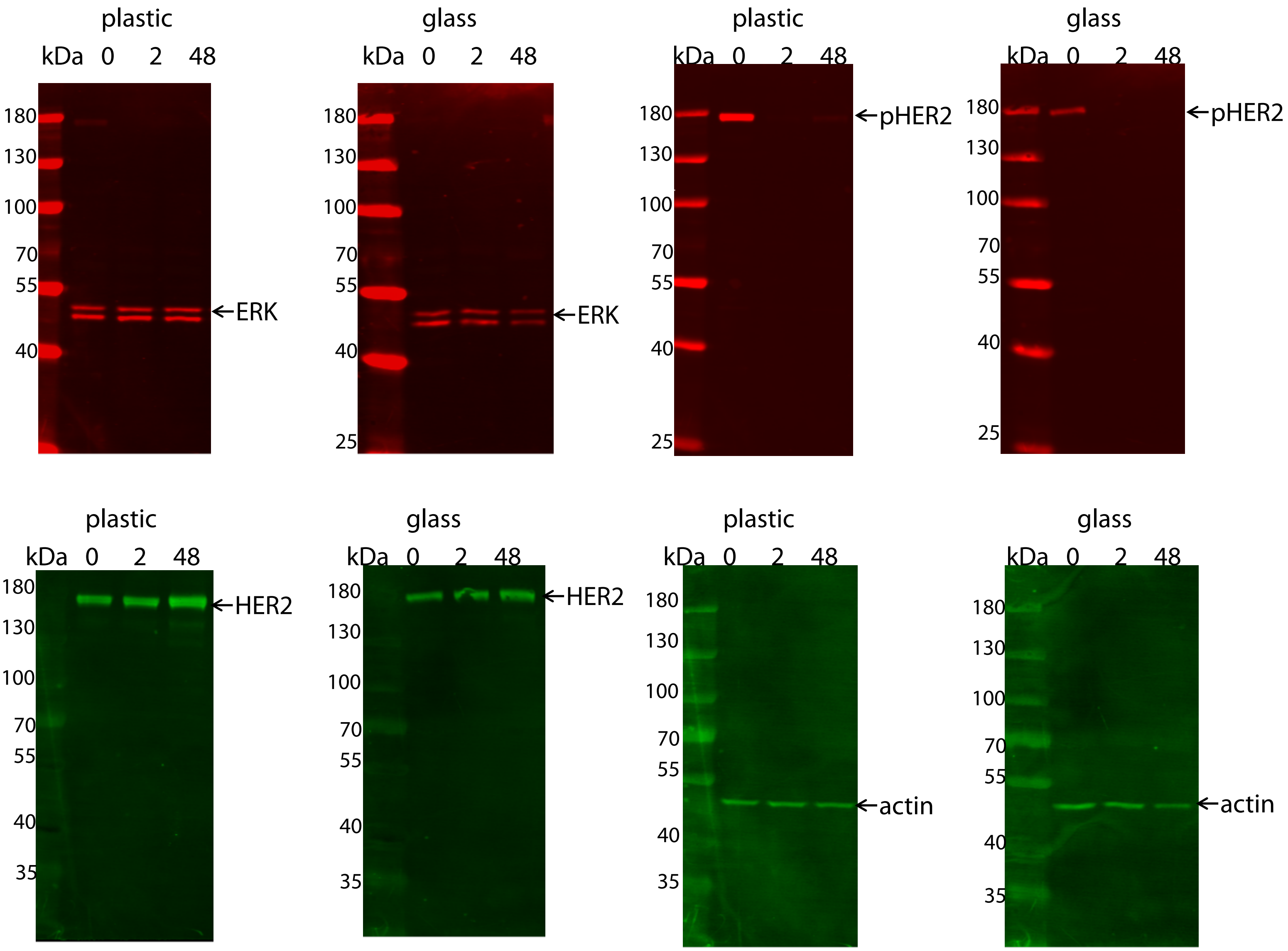

Supplement: Supplementary file 1 — Supplementary Figures [file 41598_2017_16492_MOESM1_ESM.pdf]
